# Comparison of Effects of the Ethanolic Extracts of Brazilian Propolis on Human Leukemic Cells As Assessed with the MTT Assay

**DOI:** 10.1155/2012/918956

**Published:** 2011-09-28

**Authors:** Gilberto C. Franchi, Cleber S. Moraes, Viviane C. Toreti, Andreas Daugsch, Alexandre E. Nowill, Yong K. Park

**Affiliations:** ^1^Integrated Center for Childhood Onco-Hematological Investigation, State University of Campinas, P.O. Box 6141, 13083-970 Campinas, SP, Brazil; ^2^Department of Food Science, College of Food Engineering, State University of Campinas, P.O. Box 6177, Campinas, SP, Brazil

## Abstract

Propolis is a resinous product collected by honey bees. It was also reported that propolis has a wide variety of biological actions, including antimicrobial activity and antioxidant, anti-inflammatory, and suppressive effects of dioxin toxicity activities. The aim of this study was to compare the in vitro cytotoxic activities of green propolis (G12) and red propolis (G13) in human leukemia cells. These cells were incubated with different concentrations of propolis and 48 hours after the IC_50_ was calculated for each cell. The results showed that the red propolis has cytotoxic effect in vitro higher than green propolis. Red propolis was showed to be cytostatic in K562 cells and caused the same amount of apoptosis as its control Gleevec. In conclusion, these results showed that red propolis is more cytotoxic than the green propolis in a variety of human cell lines of leukemia. Red propolis may contain drugs capable of inhibiting cancer cell growth. Therefore, further isolation of respective chemical ingredients from the red propolis (G13) for identification of the activities is necessary.

## 1. Introduction

Propolis is a resinous product collected by honey bees (*Apis mellifera*) from tree exudates mainly resins of leaf bud mixed with beeswax to form a sealing material in their honeycombs, smooth out the internal walls, and protect the entrance against intruders [1]; it was also reported that propolis has a wide variety of biological actions, including antimicrobial activity [2], antiherpes [3], and suppressive effects of dioxin toxicity [4]. Because of the wide range of biological activities, recently propolis has also been extensively used in food and beverages to improve health and diseases [5].

The medical application of propolis preparation had led to increased interest in its chemical composition and its botanical origins, because so far mainly polyphenols being flavonoids aglycones, and its derivatives. The chemical composition of the main flavonoids in propolis has been found to be quantitatively variable, depending on the environmental plant ecology [6, 7]. Therefore, we have collected 600 samples of propolis obtained by Africanized *Apis mellifera *in Brazil and then analyzed all samples. We found that Brazilian propolis is classified into 13 groups based on physicochemical characteristics. Among all groups of propolis, group 12, which is known as green propolis, is widely used mainly for ingredients of functional food and pharmaceutical purposes and the botanical origin of propolis group 12 was the resin of *Baccharis dracunculifolia* in southeastern Brazil [7]. We evaluated the effect of ethanolic extracts of the propolis group 12 and bud resins of botanical origin of propolis group 12 on proliferation of metastasis and primary tumor-derived human prostate carcinoma and observed that both samples induced growth inhibition that was associated with S phase arrest [8].

 Recently, we found reddish propolis in beehives located along the sea and river shores in northeastern Brazil. We observed that bees kept in that area were collecting the red exudates on the surface of *Dalbergia ecastaphyllum *(*L*) Taud., which is the botanical origin of propolis group 13, and both samples of the red plant exudates and the propolis were analyzed and it was found that both samples contained similar ingredients [9]. The objective of this paper was to investigate the effect of ethanolic extracts of propolis group 12 (G12) and propolis group 13 (G13) in human leukemia cells.

## 2. Materials and Methods

### 2.1. Preparation of Ethanolic Extracts of Propolis

**Figure 1:**
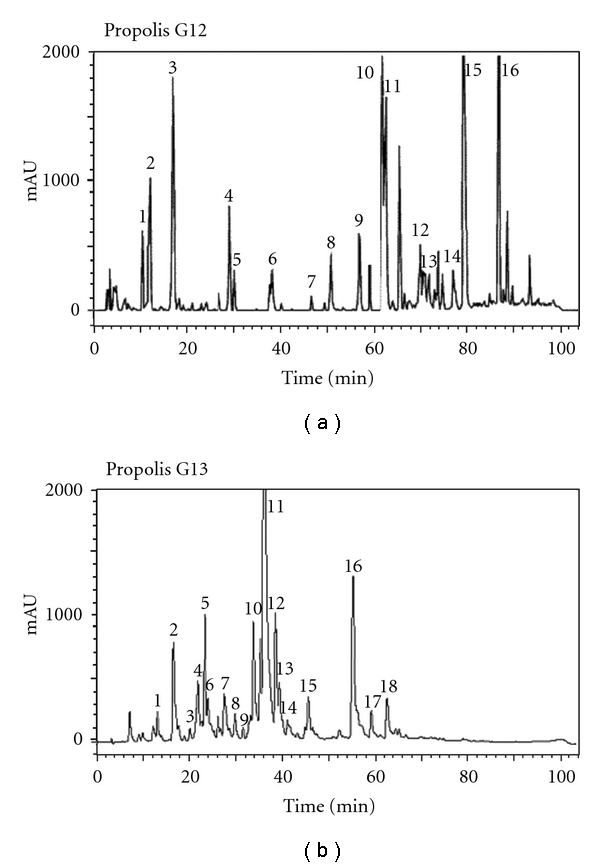
RPHPLC chromatograms of ethanolic extracts of propolis groups 12 and 13.

Recently, Brazilian propolis has been classified into 13 groups. Among these 13 groups of propolis, groups 12 and 13 were used for this investigation. Propolis group 12 was collected in the southeastern region in Brazil such as the state of São Paulo and Minas Gerais, and we have observed that bees (Africanized *Apis mellifera*) were visiting mainly bud or unexpanded leaves of *Baccharis dracunculifolia* (Compositae). In case of propolis group 13, the propolis was collected from beehives located in woody perennial shrubs along the sea and river shores in northeastern Brazil. It was observed that the bees visited mainly *Dalbergia ecastaphyllum *(*L*) Taud. (Leguminosae) to collect the red resinous exudates on its surface and from holes in the branches. Consequently, the color of propolis group 13 is also red. Two ethanolic extracts of propolis groups 12 and 13 were prepared as follows. Each group of propolis sample (50 g) was extracted with 600 mL of 80% (v/v) ethanol at 60°C for 30 min. After, extraction, the mixture was centrifuged and the supernatants were individually evaporated to complete dryness at 40°C and the resulting powder was designated as ethanolic extracts of propolis. These ethanolic extracts of propolis were analyzed by reversed-phase high-performance liquid chromatography (RPHPLC) and the results are shown in Figure 1 and Table 1. 

### 2.2. Cell Lines

The cell lines used in this study were, K562, chronic myelogenous human leukemia [10], HL60, acute promyelocytic leukemia [11], NB4, human acute promyelocytic leukemia [12], Ramos human Burkitt lymphoma [13], Raji human Burkitt lymphoma [14], Nalm16 [15] and Nalm6, human B cell precursor leukemia [16], RS4, human B cell precursor leukemia [17], B15, human B cell precursor leukemia [18], and REH, human B cell precursor leukemia [19]. The cells were grown in plastic bottles (25 cm^3^) containing RPMI 1640 (Sigma R6504) medium supplemented with 10% fetal calf serum (Gibco 16000-044), 1% penicillin (10000 IU/mL), and streptomycin (10 mg/mL) (Gibco 15070) at 37°C in humidified air with 5% CO_2_. The medium was changed every 48 h.

### 2.3. Cytotoxicity Assays

The cytotoxicity of each propolis in the cell lines indicated above was determined by the MTT (3-(4,5-dimethylthiazol-2-yl)-2-5 diphenyl tetrazolium bromide) assay. MTT is captured by cells and reduced intracellularly in a mitochondrion-dependent reaction to yield a formazan product. The ability of cells to reduce MTT provides an indication of their intactness and mitochondrial activity that serves as a measure of viability [20]. After a 48 h incubation with propolis (seven concentrations on a logarithmic scale from 1 to 1000 *μ*g/mL), the plates were centrifuged to pellet the cells, the supernatant was removed, and 10 *μ*L of MTT (Sigma, M5665) dissolved in 100 *μ*L of phosphate-buffered saline (Sigma P4417) was added followed by incubation for 4 h at 37°C in a humid, 5% CO_2_ atmosphere. After this period, the plates were centrifuged again, the supernatant was removed, and the insoluble formazan crystals were dissolved in 150 *μ*L of Isopropyl alcohol. The absorbance was read in a Synergy ELISA plate reader (Bio Tek Instruments, Highland Park, Winooski, USA) at 570 nm. The results were expressed as percentage inhibition relative to control cells (considered as 100%).

### 2.4. Trypan Blue Exclusion Test

Aliquots of K562 and Nalm16 cells (3 × 10^6^/mL, 1 mL/well) were plated in six-well culture plates (Corning, New York, NY, USA) containing RPMI 1640 medium supplemented with 10% heat inactivated fetal bovine serum (FBS) (Gibco 1600-044), 1% L-glutamine, 50 U/mL penicillin, and 50 mg/mL streptomycin, followed by the addition of group 12 or group 13 propolis and a cytotoxic reference drug. The cells were incubated in a final volume of 10 mL for 24, 48, and 72 h at 37°C in a humidified 5% CO_2_ atmosphere. At each interval, l mL of cell suspension was withdrawn and mixed with a solution of 0.4% trypan blue (Sigma T6146) to counting the cells in a Newbauer hemacytometer [21].

### 2.5. Analysis of Apoptosis by Laser Scanning Cytometry

Flow cytometry was also used to assess the cytotoxicity of the propolis extracts and cytotoxic reference drugs in each cell type [22]. The mode of cell death was analyzed using TACS Annexin V-FITC kits (R&D Systems, Inc. Minneapolis, Minn, USA). The cells were resuspended (3 × 10^6^ cells/mL) in RPMI 1640 with serum and plated in six-well polystyrene plates containing culture medium and the propolis or drug to be tested (final concentration: 100 *μ*g/mL) prior to incubation at 37°C for 1, 3, 6, 12, 24, 48, and 72 h. At the end of each period, the cells were washed once with 1 mL of PBS, centrifuged, and incubated for 15 min in medium containing calcium and annexin-V. The cells were then washed again and resuspended in 0.4 mL of buffer containing propidium iodide (5 *μ*g/mL). The samples were analyzed in a Becton Dickson FACSCanto flow cytometer in conjunction with FACSDiva software (Becton Dickson immunocytometry Systems, San Jose, Calif, USA). Control cells stained with annexin V-FITC or propidium iodide were used to adjust the cytometer compensation and gating. 

### 2.6. Statistical Analysis

Each propolis extract was screened six times against all of the cell lines and the results were expressed as mean ± standard deviation (SD). Cytotoxicity was assessed by plotting cell survival versus propolis/drug concentration (on a log scale) followed by sigmoidal curve fitting and determination of the IC_50_ by the least squares method. Boxplots were used to analyze the distribution of IC_50_ data and compare the responses to the two propolis extracts. Differences between the IC_50_ for the two propolis extracts within a given cell line were determined by using Student's *t-*test whereas differences among the IC_50_ values for a given extract among cell line were assessed by analysis of variance (ANOVA) followed by Games-Howell pos hoc test, because there was not homogeneity of variances (*P* < 0.001 for Levene's test). A value of *P* < 0.05 indicated significance. All statistical comparisons were done using SPSS software version 7.5. 

## 3. Results and Discussion

### 3.1. Cytotoxicity

**Figure 2:**
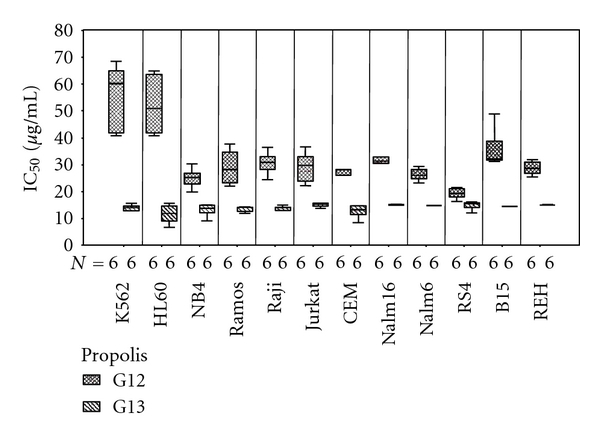
Boxplots of IC_50_ values for green propolis (G12) and red propolis (G13) in different leukemia cell lines by MTT assay.

Figure 2 (Boxplots) showed that the IC_50_ values for the two propolis extracts in the different leukemia cell lines were significantly different, with red propolis (group 13) being more potent cytotoxic in all cases. The greatest differences were observed with K562 and HL60 cells and the smallest difference with RS4 cells. Analysis of the boxplots in Figure 2 indicated that the responses to red propolis (G13) were less dispersed within each cell line and among cell lines (similar IC50 values) than those to green propolis (group 12), indicating less variation in the sensitivity of cells to the former extract. The IC_50_ values for a given extract among cell line were assessed by analysis of variance (ANOVA) followed by Games-Howell pos hoc test, because there was not homogeneity of variances (*P* < 0.001 in Levene's test for G12 and G13). ANOVA was positive for differences of IC_50_ among cell lines in both extracts (*P* < 0.001 for G12 and *P* = 0.040 for G13). These indicated that the responses to red propolis (group 13) were less dispersed within each cell line and among cell lines (similar IC_50_ values) than those to green propolis (group 12), indicating less variation in the sensitivity of cells to the former extract. The IC_50_ values for the two propolis groups were significantly different in all cases. 

**Figure 3:**
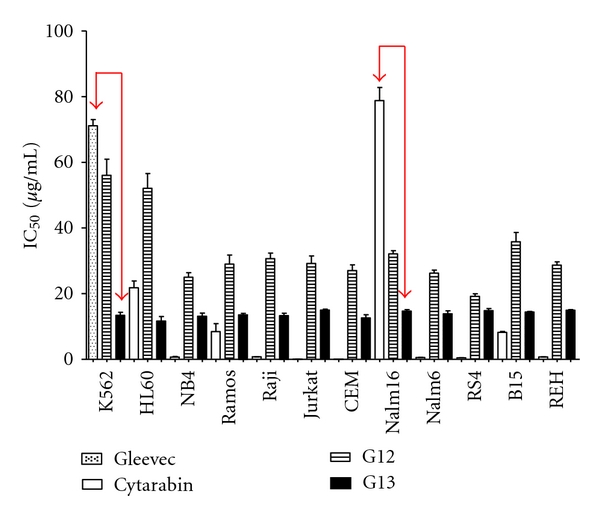
Results of MTT assay for propolis (G12) and (G13) using K562 and Nalm6.

After analyzing the data obtained from MTT, when comparing the effect of propolis G13 with Gleevec and cytarabine, there was marked difference between K562 and Nalm16 cells. We selected the two cells for testing by trypan blue exclusion and apoptosis with Annexin V. Figure 3 demonstrated that K562 and Nalm16 the biggest difference between the IC50 of G13 and cytarabine (drug used in clinical oncology) for these two cell extract of propolis G13 when the compare Gleevec or cytarabine had great significance (*P* < 0.0001). 

### 3.2. Trypan Blue Exclusion

**Figure 4:**
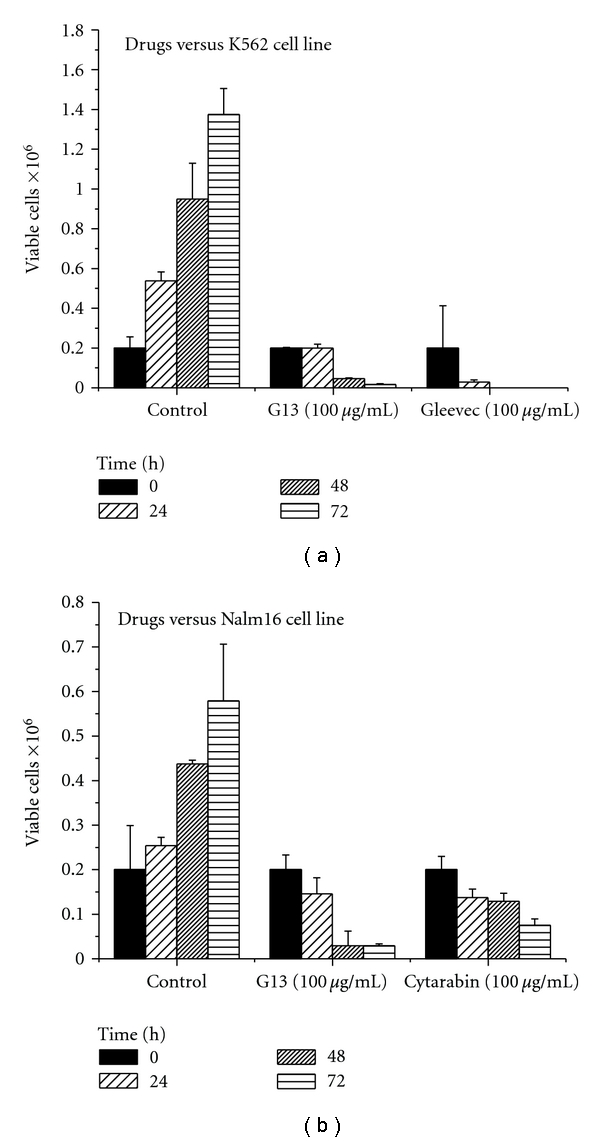
The kinetics of dye exclusion with Trypan blue.

Using trypan blue solution 0.4% (Sigma T8154), we quantified the viability of K562 cells and Nalm6 during periods of 24, 48, and 72 hours in front of G13 propolis and compared them to controls and cytarabine Gleevec. We concluded that a strong cytotoxicity reduced the number of viable K562 cells after 24 hours treating with Gleevec as shown in Figure 4(a). When we observed the effect caused by propolis G13 after 24 hours, there was a citostase because the number of viable cells is the same as the control. Observing Figure 4(b), we see a gradual reduction of cells for cytarabine Nalm16 and a great reduction in viable cells after 48 hours for G13 propolis. 

### 3.3. Apoptosis

**Figure 5:**
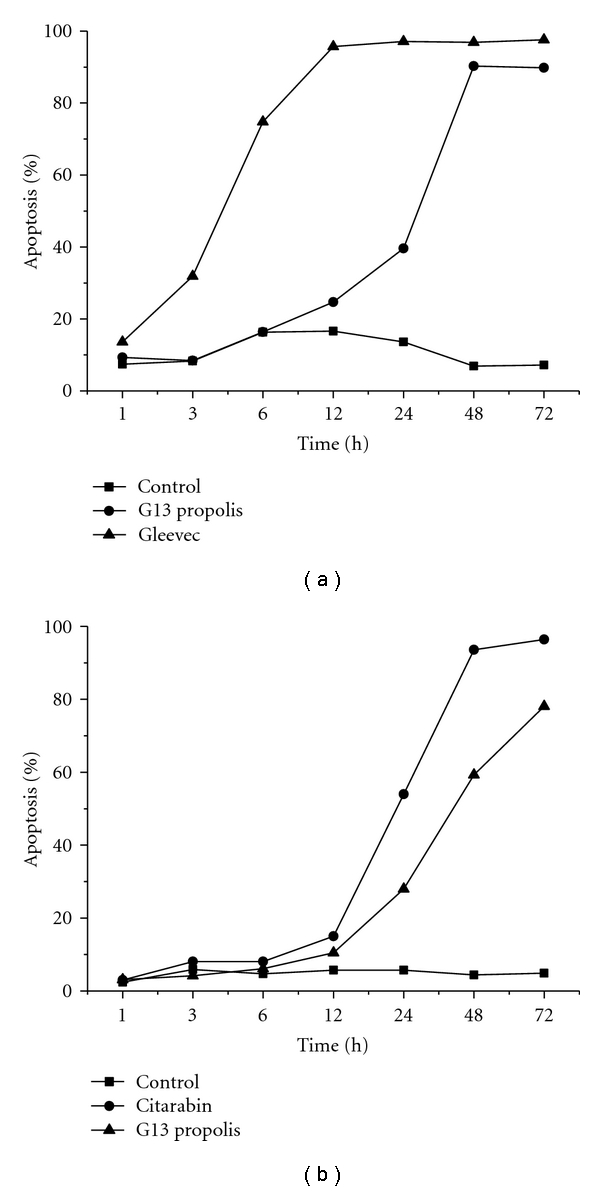
Analysis of apoptosis by laser scanning cytometry.

By Figure 5(a) it was noted that Gleevec reached the maximum apoptosis at 12 hours before the G13 propolis; this difference demonstrated the effectiveness of drug Gleevec which is the drug chosen for treatment of chronic myeloid leukemia (here represented by K562) given its efficiency; however, it was observed that the G13 could, after a period of 12 hours, reach a significant percentage of apoptosis which did not occur when comparing the cytotoxic cytarabine with propolis in Nam16 G13 cell line (Figure 5(b)). These results were obtained from a single experiment. 

## 4. Conclusion

These results indicated that propolis G13 is more cytotoxic than the green propolis (G12) in a variety of human cell lines of leukemia. G13 propolis contains chemical ingredient for inhibiting cell growth of certain types of cancer. Nalm16 and K562 cell lines, represent leukemia with high mortality, and in these trials it was suggested that there is a useful chemical ingredient in these extracts. K562 chronic myeloid leukemia is the CML in blast crisis, and the cells carry the Philadelphia chromosome with a BCR-ABL b3-a2 fusion gene (Hehlman 2007).In the past, the treatment was done with antimetabolites (cytarabine, hydroxyurea), alkylating agents, interferon alpha 2b, and steroids, but these drugs have been replaced by Gleevec. NALM-16 cell line originated from peripheral blood of a patient with relapse of leukemia pro-B lymphocyte in a subacute LLC and bad prognosis and protocolarmente; in addition to other drugs, cytarabine is used for treating this leukemia. We provide data to search for drugs with cytostatic capacity possibly present in the extract of propolis G13 raising the specter of drugs with potential therapeutic use in oncology.

## Figures and Tables

**Table 1:** Flavonoids and other chemical constituents of propolis groups 12 and 13, determined by RPHPLC (mg/g^−1^).

	Propolis G12			Propolis G13	
Peak no.	compound	Quantity in mg/g^−1^ of propolis	Peak no.	compound	Quantity in mg/g^−1^ of propolis
1	Coumaric acid	10.7	1	Rutin	1.3
2	Ferulic acid	2.4	2	Liquiritigenin	7.1
3	Λ 245 nm^a^	+	3	Daidzein	4.3
4	Cinnamic acid	2.6	4	Pinobanksin	6.0
5	Pinobanksin	1.7	5	Λ 251, 292 nm^a^	+
6	Kaempferol	1.3	6	Quercetin	1.9
7	Isosakuranetin	4.9	7	Luteolin	2.1
8	Chrysin	1.9	8	Λ 241, 272, 281 nm^a^	+
9	Acacetin	6.7	9	Dalbergin	0.9
10	Kaempferide	12.6	10	Isoliquiritigenin	12.1
11	Λ 244 nm^a^	+	11	Formononetin	19.5
12	Λ 230 nm^a^	+	12	Λ 235, 263 nm^a^	+
13	Λ 245 nm^a^	+	13	Pinocembrin	7.1
14	Λ 228, 246 nm^a^	+	14	Pinobanksin 3-acetato	2.6
15	Artepillin C	38.6	15	Biochanin A	1.5
16	Λ 223, 276 nm^a^	+	16	Λ 238, 260, 269 nm^a^	+
			17	Λ 233, 249, 329 nm^a^	+
			18	Λ 233, 256 nm^a^	+

^a^Unidentified constituents represent only UV spectral absorption maxim. + Present, but not quantified.
